# *Enterococcus faecalis* Is a Better Competitor Than Other Lactic Acid Bacteria in the Initial Colonization of Colon of Healthy Newborn Babies at First Week of Their Life

**DOI:** 10.3389/fmicb.2020.02017

**Published:** 2020-09-29

**Authors:** Mohammad Al-Balawi, Fatthy Mohamed Morsy

**Affiliations:** ^1^Biology Department, Faculty of Science, Taibah University, Medina, Saudi Arabia; ^2^Bacteriology Section, Botany and Microbiology Department, Faculty of Science, Assiut University, Assiut, Egypt

**Keywords:** *Enterococcus faecalis*, *Lacticaseibacillus rhamnosus*, *Limosilactobacillus reuteri*, newborn babies, stool, initial colonization

## Abstract

Initial colonization of human gut by bacteria is an important step in controlling its microbiota and health status. This study followed the initial colonization by lactic acid bacteria (LAB) in colon of new born babies through following its occurrence in their stool at first week of their life. The LAB occurrence in the neonates’ stool was followed on MRS agar medium. The isolated LAB from male and female newborn babies of normal birth and cesarean section surgical delivery were molecular biologically identified by phylogenetic analysis of 16S rRNA gene sequence. From the 24 investigated newborn babies, three LAB taxa, *Lactobacillaceae*, *Enterococcus*, and *Streptococcus*, were detected in their stool at first week of their life. *Lactobacillaceae* represented 20.8% of total colonized LAB in newborn babies in the culture-dependent approach used in this study and included three species namely *Limosilactobacillus reuteri* (previously known as *Lactobacillus reuteri*), *Lacticaseibacillus rhamnosus* (previously known as *Lactobacillus rhamnosus*) and *Ligilactobacillus agilis* (previously known as *Lactobacillus agilis*). *Enterococcus faecalis* and *E. faecium* were detected where *E. faecalis* was the highest dominant, representing 62.5% of total LAB colonizing newborn babies. This result suggests that this bacterium has high potency for colonization and might be important for controlling the initial settlement of microbiota in healthy newborn babies. Only one species of *Streptococcus* namely *Streptococcus agalactiae* was detected in 8.33% total of the investigated newborn babies indicating high competency by other LAB for colonization and that this bacteria, in spite of its pathogenicity, is commensal in its low existence in healthy babies. The explored potency of natural initial colonization of the LAB species *E. faecalis*, *E. faecium*, *L. reuteri*, *L. rhamnosus*, and *L. agilis* of which many health beneficial strains were previously reported, would be important for future applications. Despite the controversy in evaluating its health benefits, *E. faecalis* as a potent competitor to other LAB refers to its importance in initial colonization of healthy babies colon at first week of their life. Further future studies, with more number of samples and characterization, would be of importance for evaluating the potential use of beneficial *Enterococcus* strains which could improve intestinal ecosystem.

## Highlights

- Initial colonization of healthy 24 newborn babies by various lactic acid bacteria was followed.
- Three genus of lactic acid bacteria *Lactobacillus*, *Enterococcus*, and *Streptococcus* were detected.
- The species *Lactobacillus reuteri*, *Lactobacillus rhamnosus*, and *Lactobacillus agilis* were found.
- All *Lactobacillus* species represented as a whole 20.8% of total colonized lactic acid bacteria in newborn babies.
- *Enterococcus faecalis* represented 62.5% of total lactic acid bacteria colonized newborn babies.

## Introduction

The initial bacterial colonization of human gut after birth is of importance for the control of microbiota settlement and for many health benefits. Microbiota colonizing the gut helps in driving post-natal maturation of the developing infant gut and plays also a role in of the mucosal immune system development (Gronlund et al., 2000; Hooper and Gordon, 2001; Hooper et al., 2002; Macpherson and Harris, 2004; Sjögren et al., 2009; Livingston et al., 2010; Donovan et al., 2012; Brugman et al., 2015; Timmerman et al., 2017). Some strains of LAB are able to promote intestinal health of their hosts (Lionetti et al., 2006; Ma et al., 2009; Jones et al., 2012; Kechagia et al., 2013; Mackos et al., 2016; Linares et al., 2017). Some strains of *Limosilactobacillus reuteri* (previously known as *Lactobacillus reuteri*; Zheng et al., 2020) is known for its beneficial roles in health (Mackos et al., 2013; Keller et al., 2014; Hou et al., 2015; Lee et al., 2016; Hsu et al., 2017; Mu et al., 2018), colonizing the human and animal intestine and producing the antimicrobial compound reuterin (Talarico and Dobrogosz, 1989; Cadieux et al., 2008; Morita et al., 2008; Spinler et al., 2008; Schaefer et al., 2010; Mishra et al., 2012; Mu et al., 2018; Asare et al., 2020; Senatore et al., 2020) which can inhibit the growth of several Gram-positive and Gram-negative bacteria (Cleusix et al., 2007). It has also been shown that some strains of *Lacticaseibacillus rhamnosus* (previously known as *Lactobacillus rhamnosus*; Zheng et al., 2020) have beneficial roles in health promotion (Martinez et al., 2009; Allonsius et al., 2017; George-Kerry et al., 2018; Westerik et al., 2018a,b).

Application of molecular identification tests along with metagenomic analysis for profiling the human colon microbiota (Qin et al., 2010; Maccaferri et al., 2011; Wang et al., 2015; Almeida et al., 2019; Franco-Duarte et al., 2019) would be of importance for diagnosing the health development of newborn babies. This would help in better understanding the colonization and various roles of colon beneficial bacteria for human health. The cost of molecular identification tests of microbes is continuously ongoing to be the cheapest. Along with its accuracy and fastness (Franco-Duarte et al., 2019), molecular identification tests would be the best choice not only for research purposes but for applicable gut health status diagnosis in near future. Thus, profiling the normal colon flora including the health beneficial bacterial species though molecular identification tests along with metagenomic analysis is a hot topic that would enrich the data base for following up the health development in newborn babies.

The health beneficial roles and use of *Enterococcus* strains is a controversial topic. The health benefits of specific strains of *Enterococcus faecalis* and *Enterococcus faecium* were characterized (Hlivak et al., 2005; Franz et al., 2011; Cebrián et al., 2012; Hanchi et al., 2018; Zommiti et al., 2018). Some strains of *E. faecalis* and *E. faecium* and some others strains belonging to the species *Enterococcus durans*, *Enterococcus hirae*, *Enterococcus lactis*, and *Enterococcus munditii* had the potential to promote health to their hosts (Nami et al., 2014; Pieniz et al., 2014; Nami et al., 2015; Gupta and Tiwari, 2015; Haghshenas et al., 2016; van Zyl et al., 2016). However, extensive studies for safety regulation is required upon application. Following the occurrence of these species of *Enterococcus* and other LAB in the human gut in newborn healthy babies, would explore its potency for colonization and possible roles at the early stage of life and initial establishment of gut microbiome. In this study, the occurrence of cultivable LAB in newborn infants’ stool was followed at the first week of their life for exploring its potency for initial colonization of healthy newborn babies colon.

## Materials and Methods

Colonization of LAB at first week of life of newborn babies was followed by following the occurrence of theses bacteria in newborn infants’ stool in Almadinah Almunawarah, Kingdom of Saudi Arabia (KSA).

### Sampling and LAB Isolation

LAB occurrence in stool of healthy newborn babies in the first week of their life (age 2–6 days old) was investigated. The stool of 24 newborn babies were sampled, 12 from who had normal delivery and 12 from who had cesarean birth. In each of these groups, six newborn babies were girls and six were boys. The healthy newborn babies were selected randomly. LAB occurrence in stool of healthy newborn babies was investigated using de Man, Rogosa, and Sharpe agar (MRS agar, HiMedia Laboratories Limited, Mumbai, India)]. Serial dilution of stool samples was conducted and subsequently LAB were detected on the MRS medium after 48 h of inoculation and incubation in CO_2_ incubator (5% CO_2_) at 35°C and colony forming units (CFU) was followed by most probable number (MPN) techniques (MacFaddin, 1985). The MRS plates with low detectable number of colonies were used for determining the CFU of dominating colonies with similar morphological and bacterial biochemical characteristics of which an isolated strain was subcultured in MRS agar to obtain a purified strain.

### Specie-Level Identification of LAB Strains

The specie-level identification of LAB strains was conducted by phylogenetic analysis of 16S rRNA encoding gene sequence. The bacterial strains were cultured in MRS broth medium for 48 h for genomic DNA extraction.

#### DNA Isolation and 16S rRNA Encoding Gene Amplification

Bacterial cells from 1 mL culture of each LAB strain were collected by centrifugation and used for genomic DNA extraction. The DNA extraction was conducted using the Promega Wizard Genomic DNA Purification Kit (Promega Corporation, Madison, Wisconsin, United States) following the kit manufacturer instructions. The extracted genomic DNA of each LAB strain was subsequently used as a template for PCR amplification of 16S rRNA encoding gene.

The PCR amplification of 16S rRNA encoding gene was conducted using the universal 27F forward and 1492R reverse primers where the sequence of the 27F forward primer was (5′-AGAGTTTGATC[A/C]TGGCTCAG-3′) and the sequence of the 1492R reverse primer was (5′-G[C/T]TACCTTGTTACGACTT-3′) (Lane, 1991). A near-full length amplification by PCR of the 16S rRNA encoding gene was performed with a reaction mixture (25 μl) composed by 10× *Taq* buffer (100 mM Tris-HCl, pH 8) 2.5 μl; MgCl_2_ (1.25 mM); dNTPs (100 μM) (Invitrogen, Carlsbad, CA, United States); forward and reverse primer (1.2 μM); *Taq* DNA polymerase (Invitrogen, United States) (0.5 U) and the bacterial genomic DNA as a template of about 5 ng. PCR was performed using a Thermal Cycler (Model 2720; Applied Biosystems, Foster City, California, United States). PCR program was implemented as follows: initial denaturation step at 95°C for 5 min followed by 35 amplification cycles of [94°C for 1 min (denaturation); 56°C for 1 min (annealing); 72°C for 1 min (extension)] and a subsequent final extension step at 72°C for 10 min. Analyzing the PCR amplification products was conducted using agarose electrophoresis on agarose (1%) gels containing 5 μg/mL ethidium bromide with using a DNA size marker [(Invitrogen, United States) 1 kb Plus DNA ladder].

#### Sequencing and Sequence Analysis

The PCR products were purified and subsequently cycle sequenced at Macrogen Korea sequencing facility (Seoul, Korea). Direct cycle sequencing using same forward and reverse primers in both directions was performed for sequencing the PCR purified product using automated florescent dye terminator sequencing method (Sanger et al., 1977) with DNA Analyzer 3730XL (Applied Biosystems, CA, United States) at Macrogen Korea sequencing facility (Seoul, Korea). The sequence reads of the 16S rRNA gene sequence with forward and the reverse primers were assembled and compared with closest matches of DNA sequences found in GenBank using the search tools of nucleotide-nucleotide BLAST at www.ncbi.nlm.nih.gov/blast/Blast.cgi in the NCBI (National Center for Biotechnology and Information) website. The alignments of the 16S rRNA gene sequences were done according to Thompson et al. (1997) by Clustal W1.83 XP. Using neighbor-joining method (Saitou and Nei, 1987), the phylogenetic trees of 16S rRNA encoding gene sequences were constructed with MEGAX software (Kumar et al., 2018). An outgroup [*Bacillus subtilis* strain JCM 1465; accession number (NR_113265)] was used.

### Nucleotide Sequences Accession Numbers

The 16S rRNA encoding gene partial nucleotide sequence of each LAB strain was deposited under an accession number for each in the GenBank nucleotide sequence database. The accession numbers of the deposited 16S gene sequence of all LAB strains are outlined in Table 1.

**TABLE 1:** Lactic acid bacterial strains detected in stool of newborn babies from various delivery modes (normal birth and surgical delivery by cesarean section) at first week of life (age 2–6 days old).

#	Mode of delivery	Bacterial isolates	Strain	Accession number
1	NB^*a*^	*Lactobacillus agilis*	TEM 13	MT525311
2	NB	*Enterococcus faecalis*	TEM 6	MT533849
3	NB	*Enterococcus faecalis*	TEM 8	MT533852
4	NB	*Streptococcus agalactiae*	TEM 5	MT527194
5	NB	*Enterococcus faecalis*	TEM 10	MT534027
6	NB	*Enterococcus faecalis*	TEM 11	MT534011
7	NB	*Enterococcus faecalis*	TEM 4	MT526391
8	NB	*Enterococcus faecalis*	TEM 9	MT533885
9	NB	*Enterococcus faecalis*	TEM 17	MT539109
10	NB	*Enterococcus faecalis*	TEM 20	MT539117
11	NB	*Enterococcus faecalis*	TEM 73	MT539132
12	NB	*Enterococcus faecalis*	TEM 75	MT539138
13	SD^*b*^	*Lactobacillus reuteri*	TEM 69	MT521084
14	SD	*Lactobacillus reuteri*	TEM 70	MT525281
15	SD	*Lactobacillus rhamnosus*	TEM 67	MT250507
16	SD	*Lactobacillus rhamnosus*	TEM 80	MT516387
17	SD	*Streptococcus agalactiae*	TEM 14	MT527545
18	SD	*Enterococcus faecium*	TEM 1	MT525359
19	SD	*Enterococcus faecium*	TEM 81	MT539118
20	SD	*Enterococcus faecalis*	TEM 15	MT535540
21	SD	*Enterococcus faecalis*	TEM 16	MT539106
22	SD	*Enterococcus faecalis*	TEM 18	MT539112
23	SD	*Enterococcus faecalis*	TEM 19	MT539113
24	SD	*Enterococcus faecalis*	TEM 68	MT539119

**^*a*^NB*, *Normal Birth.**

*^b^SD*, *Surgical Delivery by cesarean section.*

## Results

In this study, the occurrence of LAB in newborn infants’ stool was followed to explore the colonization of various LAB in colon of newborn babies at first week of their life. All of the bacterial strains isolated from the stool of the 24 newborn babies were identified by phylogenetic analysis of 16S gene sequence. From all of the newborn babies subjects investigated for colonization by LAB, three LAB genus namely *Enterococcus*, *Lactobacillus*, and *Streptococcus* were detected (Table 1) in stool of newborn subjects at this stage of initial colonization in this start of babies life. The isolated LAB species was dominant in each case of the 24 healthy newborn babies which is probably because the colonization is initial at the first week of the babies life.

**FIGURE 1:**
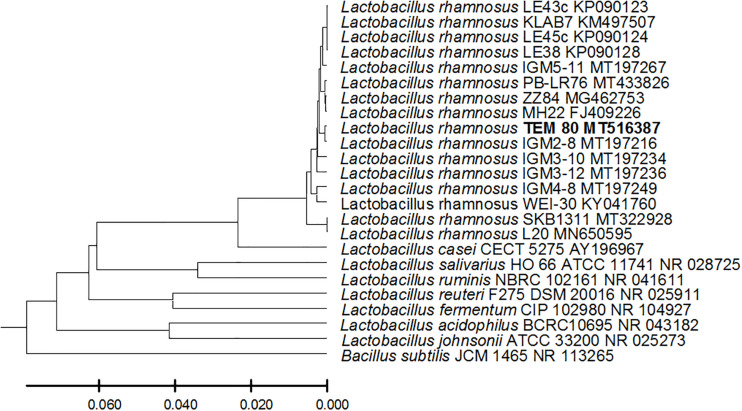
*Lactobacillus* rhamnosus strain TEM 80 16S rRNA encoding gene phylogenetic analysis. Phylogenetic tree of *Lactobacillus rhamnosus* strain TEM 80 isolated from newborn infants’ stool showing relationship with its closest bacterial neighbors strains from NCBI. The *Lactobacillus rhamnosus* strain TEM 80 is shown in bold. The neighbor-joining tree of isolated *Lactobacillus rhamnosus* strain TEM 80 and other bacterial strains was determined using 16S rRNA encoding gene sequences and the frequency filter in MEGA-X analysis package software. An out group *Bacillus subtilis* strain JCM 1465 (accession number; NR_113265) was used. A scale bar segment indicates 2% estimated difference in sequence. Each strain accession number in the NCBI database is shown.

**FIGURE 2:**
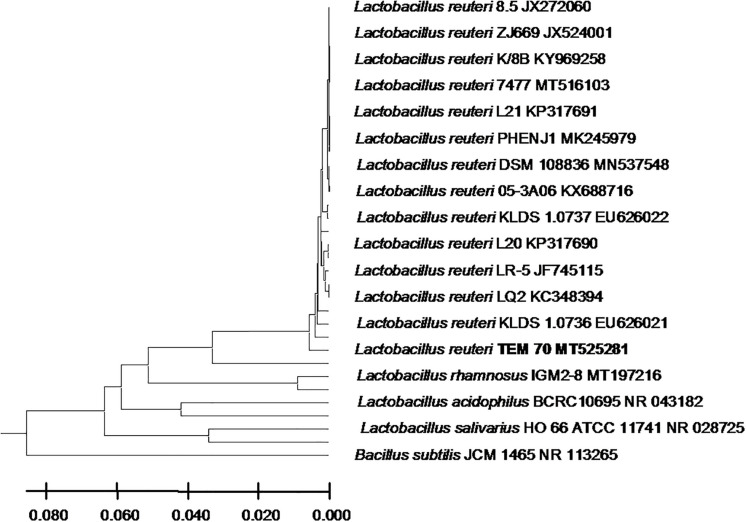
*Lactobacillus reuteri* strain TEM 70 16S rRNA encoding gene phylogenetic analysis. Phylogenetic tree of *Lactobacillus reuteri* strain TEM 70 isolated from newborn infants’ stool showing relationship with its closest bacterial neighbors strains from NCBI. The *Lactobacillus reuteri* strain TEM 70 is shown in bold. The neighbor-joining tree of isolated *Lactobacillus reuteri* strain TEM 70 and other bacterial strains was determined using 16S rRNA encoding gene sequences and the frequency filter in MEGA-X analysis package software. An out group *Bacillus subtilis* strain JCM 1465 (accession number; NR_113265) was used. A scale bar segment indicates 2% estimated difference in sequence. Each strain accession number in the NCBI database is shown.

**FIGURE 3:**
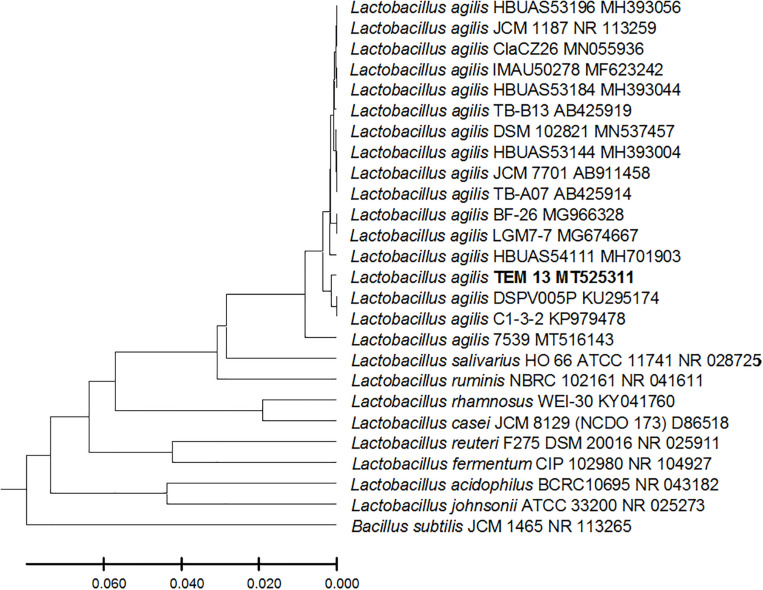
*Lactobacillus agilis* strain TEM 13 16S rRNA encoding gene phylogenetic analysis. Phylogenetic tree of *Lactobacillus agilis* strain TEM 13 isolated from newborn infants’ stool showing relationship with its closest bacterial neighbors strains from NCBI. The *Lactobacillus agilis* strain TEM 13 is shown in bold. The neighbor-joining tree of isolated *Lactobacillus agilis* strain TEM 13 and other bacterial strains was determined using 16S rRNA encoding gene sequences and the frequency filter in MEGA-X analysis package software. An out group *Bacillus subtilis* strain JCM 1465 (accession number; NR_113265) was used. A scale bar segment indicates 2% estimated difference in sequence. Each strain accession number in the NCBI database is shown.

**FIGURE 4:**
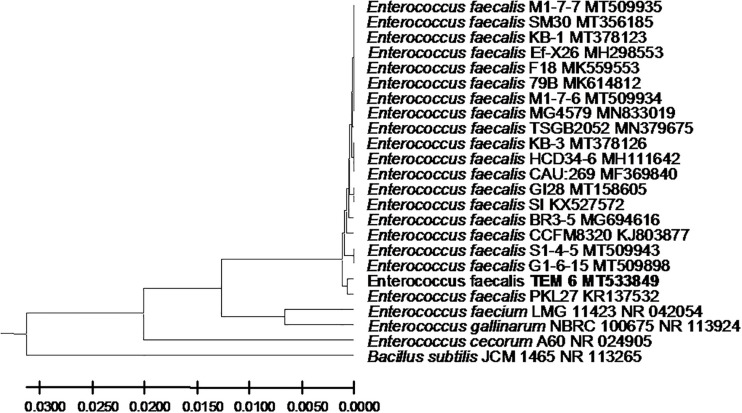
*Enterococcus faecalis* strain TEM 6 16S rRNA encoding gene phylogenetic analysis. Phylogenetic tree of *Enterococcus faecalis* strain TEM 6 isolated from newborn infants’ stool showing relationship with its closest bacterial neighbors strains from NCBI. The *Enterococcus faecalis* strain TEM 6 is shown in bold. The neighbor-joining tree of isolated *Enterococcus faecalis* strain TEM 6 and other bacterial strains was determined using 16S rRNA encoding gene sequences and the frequency filter in MEGA-X analysis package software. An out group *Bacillus subtilis* strain JCM 1465 (accession number; NR_113265) was used. A scale bar segment indicates 2% estimated difference in sequence. Each strain accession number in the NCBI database is shown.

**FIGURE 5:**
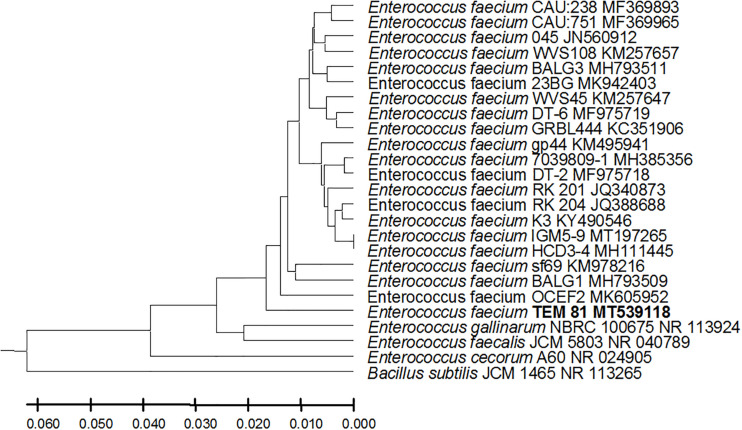
*Enterococcus faecium* strain TEM 81 16S rRNA encoding gene phylogenetic analysis. Phylogenetic tree of *Enterococcus faecium* strain TEM 81 isolated from newborn infants’ stool showing relationship with its closest bacterial neighbors strains from NCBI. The *Enterococcus faecium* strain TEM 81 is shown in bold. The neighbor-joining tree of isolated *Enterococcus faecium* strain TEM 81 and other bacterial strains was determined using 16S rRNA encoding gene sequences and the frequency filter in MEGA-X analysis package software. An out group *Bacillus subtilis* strain JCM 1465 (accession number; NR_113265) was used. A scale bar segment indicates 2% estimated difference in sequence. Each strain accession number in the NCBI database is shown.

**FIGURE 6:**
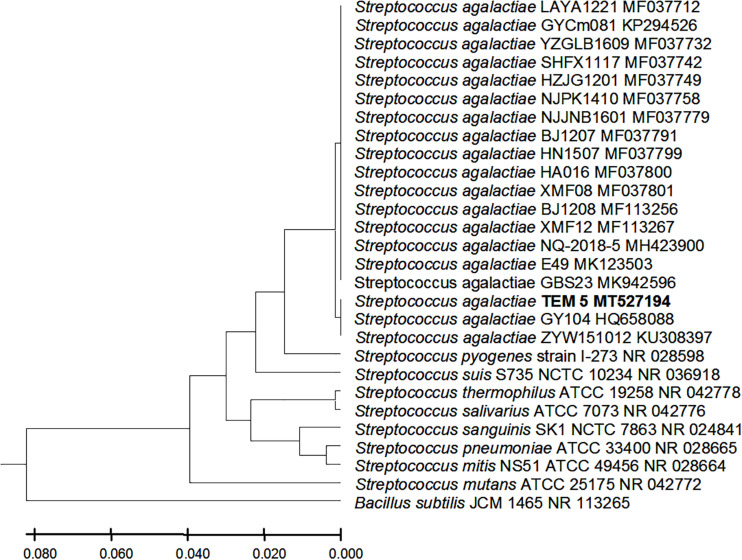
*Streptococcus agalactiae* strains TEM 5 16S rRNA encoding gene phylogenetic analysis. Phylogenetic tree of *Streptococcus agalactiae* strains TEM 5 isolated from newborn infants’ stool showing relationship with its closest bacterial neighbors strains from NCBI. The *Streptococcus agalactiae* strains TEM 5 is shown in bold. The neighbor-joining tree of isolated *Streptococcus agalactiae* strains TEM 5 and other bacterial strains was determined using 16S rRNA encoding gene sequences and the frequency filter in MEGA-X analysis package software. An out group *Bacillus subtilis* strain JCM 1465 (accession number; NR_113265) was used. A scale bar segment indicates 2% estimated difference in sequence. Each strain accession number in the NCBI database is shown.

**FIGURE 7:**
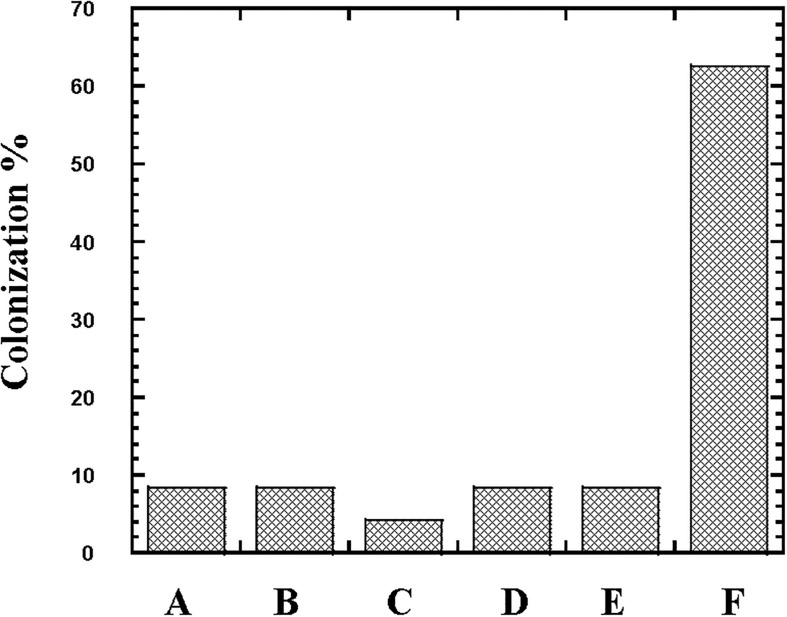
Colonization (%) of the lactic acid bacteria *Lactobacillus, Streptococcus* and *Enterococcus* in newborn babies subjects. The occurrence of *Lactobacillus reuteri* (Column A), *Lactobacillus rhamnosus* (Column B), *Lactobacillus agilis* (Column C), *Streptococcus agalactiae* (Column D), *Enterococcus faecium* (Column E), and *Enterococcus faecalis* (Column F) in stool of 24 newborn babies subjects was followed at first week of their life.

From the 24 babies, two subjects retained *L. rhamnosus* which represent 8.33% of the total LAB occurrence in all of the 24 investigated newborn subjects. The 16S rRNA encoding gene of the isolated *L. rhamnosus* strain TEM 67 and strain TEM 80 was sequenced and submitted to gene bank for accession number (Table 1). Phylogenetic analysis of the 16S rRNA encoding gene sequence was conducted for identification of *L. rhamnosus* (Figure 1). Two other species of *Lactobacillus* were detected namely *L. reuteri* and *L. agilis. Limosilactobacillus reuteri* was detected in stool samples of two newborn subjects representing a similar percent of occurrence to *L. rhamnosus* of the total LAB in all investigated newborn subjects. Phylogenetic analysis of *L. reuteri* of the 16S rRNA encoding gene sequence was conducted (Figure 2). *Ligilactobacillus agilis* was the most rare of all LAB detected out the 24 investigated newborn subjects where it represented 4.16% of the total LAB occurrence. This bacterial strain was found in stool a newborn baby of normal birth vaginal delivery, suggesting that vaginal delivery normal birth process might be the source of the inoculation by this bacterium to newborn baby through vaginal birth canal. The molecular biological identification by phylogenetic analysis for the isolated *L. agilis* strain TEM 13 is shown (Figure 3). The colonization of newborn babies colon by the three species of *Lactobacillaceae* (*L. rhamnosus*, *L. reuteri*, and *L. agilis*) at this early stage of life indicate its importance for the healthy babies where several strains of theses species was previously reported as health beneficial ones. The two bacterial species with controversial hypothesized health benefits of *Enterococcus* namely *E. faecalis* and *E. faecium* were detected where *E. faecalis* was the most abundant bacterium detected in babies subjects. Phylogenetic analysis tree of 16S rRNA gene sequence for molecular biological identification of *E. faecalis* and *E. faecium* is shown in Figures 4, 5, respectively. The occurrence and molecular characterization of LAB in newborn infants’ stool in Al-Madinah, KSA reveals higher potency of the facultative anaerobes *E. faecalis* for earlier colonization of healthy babies colon than other detected LAB. *Enterococcus* appeared in newborn infants’ stool whatever the mode of delivery or gender (Table 1) indicating a high potency of this facultative anaerobic bacterium to colonize babies gut at this early stage of life. The results in this study on healthy newborn babies might supports the hypothesis that *E. faecalis*, of which some health beneficial strains were reported, can be used with potential initial colonization as this specie naturally and potentially colonizes healthy babies colon as appeared in their stool at the first week of their life with no disorder syndrome suggesting its compatibility for a healthy conditions in human gut and for the colon microbiome initial settlement at the start of human life. *Streptococcus* genus existence was low compared to total LAB and only one species namely *S. agalactiae* was found (Table 1) and identified by 16S phylogenetic analysis (Figure 6). The low occurrence of *S. agalactiae* in stool of healthy newborn babies subjects (Figure 7) compared to total LAB indicates high competition by other LAB *Enterococcus* and *Lactobacillus* against colonization by this *S. agalactiae* pathogenic bacterium. In spite of its rare existence, the detection of the pathogenic bacterium *S. agalactiae* in healthy newborn babies might also indicate its commensal status.

## Discussion

The health beneficial properties of some strains of the LAB *E. faecalis*, *L. reuteri*, *L. rhamnosus, L. agilis*, and others were previously reported. This study was devoted for investigating the initial colonization of healthy newborn babies colon at the first week of their life by various LAB through following its occurrence in the neonates’ stool. The study targeted to follow up the potency of colonization by these LAB species of which many strains health beneficial properties were previously reported where investigating the initial colonization potency of these LAB species explored in this study is important to know for assessment of the usage efficiency of these LAB species in applications.

In this study, the high tendency for colonization of newborn gut by *E. faecalis* at this early stage of life and initial formation of the gut microbiome refers to its efficiency in colonization. In each case of the 24 healthy newborn babies, the isolated LAB species was dominant. This is probably because the colonization is initial and hence *E. faecalis*, representing 62.5% of total LAB colonizing healthy newborn babies, is shown as a better competitor than other LAB in the initial colonization. Because of their tolerance to wide range of temperature and pH, many *Enterococcus* species are highly competitive (Hanchi et al., 2018). The only *Enterococci* yet suggested to be used for health benefits are some specific strains of *E. faecalis* and *E. faecium* (Franz et al., 2011; Cebrián et al., 2012). Such high competitiveness *E. faecalis* for colonization can be also attributed to their ability to produce bacteriocins (Cebrián et al., 2012; Hanchi et al., 2018) that would support the potency of colonization of *Enterococcus* against other bacteria. This ability to effectively colonize the gut of newborn babies by *E. faecalis* at this early stage of life might be related to other factors including its facultative anaerobiosis metabolism where a successful colonization by *Lactobacillaceae* and other microaerobes or anaerobes in the human gut would require installation of microaerobic and anaerobic conditions in the lumen of the colon. Such installation of microaerobic conditions might require time and varies from a newborn subject to the other at this initial stage of colonization. The facultative anaerobes including *Enterococcus* and the widely spread *Escherichia coli* would play important role in consuming molecular oxygen for installing microaerobic and anaerobic conditions suitable for vitality and possible later enriched colonization by *Lactobacilli* and other microaerobic and anaerobic bacteria where aerobic and facultative anaerobic bacterial consumption of oxygen along with other mechanisms of O_2_ consumption was suggested to maintain the lumen of the gut in a deeply anaerobic state that is important for obligate anaerobes (Friedman et al., 2018). Colonization by *L. rhamnosus*, *L. reuteri*, and *L. agilis* detected in only around 20% of total of the newborn babies subjects indicates the possibility of variation in ability to install the microaerobic and anaerobic conditions suitable for viability of these bacteria and its potential of colonization. The high potency for colonization of *E. faecalis* in healthy newborn babies might prevent colonization by virulent pathogenic bacteria possibly through its ability to produce antimicrobial agents bacteriocins (Franz et al., 2011; Hanchi et al., 2018). In addition to *L. reuteri* which is known to produce the antimicrobial reuterin (Talarico et al., 1988; Spinler et al., 2008; Jones and Versalovic, 2009; Greifova et al., 2017; Mu et al., 2018; Asare et al., 2020; Senatore et al., 2020) these bacteria in healthy newborn babies might act as defenders against colonization by virulent pathogenic bacteria in this early stage of life. While the antimicrobial reuterin produced by *L. reuteri* can inhibit other microorganisms (Cleusix et al., 2007) that can help in controlling the initial microbial colonization in newborn babies to reduce the possible colonization by pathogenic bacteria. Many other benefits of *L. reuteri* includes reduction of infections by harmful bacteria, decreasing bacterial translocation, promoting health, increasing the absorption of minerals, vitamins and nutrients, modulating the immune responses of the host, and promoting the integrity of gut mucosa (Wang et al., 1995; Adawi et al., 1997; Nikawa et al., 2004; Niv et al., 2005; Saggioro et al., 2005; Tubelius et al., 2005; Imase et al., 2007; McFall-Ngai, 2007; Francavilla et al., 2008; Indrio et al., 2008; Spinler et al., 2008; Hou et al., 2015; Mu et al., 2018). Controlling the initial microbiotal colonization in newborn babies can also be aided by *L. rhamnosus* which appeared in stool of newborn babies in similar detection percent to *L. reuteri* and is also well-known for its healthy effects (Szajewska et al., 2015; Urbańska et al., 2016; Allonsius et al., 2017; Westerik et al., 2018a,b). The colonization with *S. agalactiae* (Group B *Streptococcus*) was low in stool of healthy newborn babies subjects. *Streptococcus agalactiae* is commensal bacterium in the human intestinal and genitourinary tracts (Kaambo et al., 2018) where in healthy adults, this Group B *Streptococcus* rarely cause infections; however, it may occasionally cause morbidity in pregnant women (Sørensen et al., 2010; Brigtsen et al., 2015; Kaambo et al., 2018) and is a major risk factor in newborns for Group B *Streptococcus* early onset invasive disease (GBS EOD) (Kwatra et al., 2014; Brigtsen et al., 2015; Kaambo et al., 2018). The rare colonization by this bacterium indicates that other dominant LAB might hinders its colonization. Such rare existence of this bacterium in healthy newborn babies might also indicate that this commensal bacterium is not harmful except in case of overgrowth which would be hindered by other LAB such as *L. reuteri* and *E. faecalis* producing antimicrobial agents. The antagonistic effect of *Lactobacilli* against *S. agalactiae* is well-documented (Ho et al., 2016; Sharpe et al., 2019). *Streptococcus agalactiae* is well-known as a commensal member of the vaginal microflora (El Aila et al., 2009; Meyn et al., 2009; Sangeetha et al., 2015; Kaambo et al., 2018; Shabayek and Spellerberg, 2018) suggesting that vaginal canal is the source of inoculation in newborn babies of vaginal delivery normal birth. However, the bacterium colonized not only newborn babies of vaginal delivery normal birth but also babies of cesarean section surgical delivery, indicating that inoculation might also occur from surrounding environments or during feeding process. The third species of *Lactobacilli* appeared in stool of newborn baby of normal birth vaginal delivery was *L. agilis*. This *Lactobacilli* was previously isolated from human vagina (Ocana et al., 1999) suggesting that inoculation of newborn babies by this bacterium might occur during normal delivery through the vagina. Some strains of *L. agilis* was suggested as health beneficial strains that can be applied as a feed additive for poultry (Lan et al., 2003; Ren et al., 2019) and was considered as one of the vaginal *Lactobacilli* potential antagonistic against *Candida* spp. (Gil et al., 2010). Thus, this bacterium can also act for minimizing the colonization of newborn babies colon by pathogenic bacteria through competing for the colonization space in addition to other antimicrobial agents producing *L. reuteri*, *E. faecalis*, and *E. faecium.*

In near future, the use of molecular identification tests with the broadening gene bank data base would be of importance for applicable diagnosis of bacterial flora in newborn babies for following up their health development status. In addition, the metagenomic analysis for profiling the whole microbiota in colon would be very helpful for achieving this applicable future diagnosis target of health development assessments including the initial colonization of the colon by beneficial bacterial species.

In conclusion, the results in this study showed that *E. faecalis* was most dominant representing more than 60% of the total LAB in stool of healthy newborn babies and is hence a better competitor to other LAB including *L. reuteri* and *L. rhamnosus* in the initial colonization of healthy babies colon at first week of their life. These results in healthy babies suggest possible beneficial role of this bacterium in addition to the detected *Lactobacilli* for minimizing colonization by pathogenic LAB such as *S. agalactiae* which showed minimum initial colonization of newborn babies at the first week of their life.

## Data Availability Statement

The datasets generated for this study can be found in the online repositories. The names of the repository/repositories and accession number(s) can be found in the article/supplementary material.

## Ethics Statement

The studies involving human participants were reviewed and approved by the Institutional Review Board (IRB) in Medina [Head of IRB Committee (Abduhameed Alsubhi)]. Informed consent was obtained from parents for the collection of infant stools.

## Author Contributions

All authors listed have made a substantial, direct and intellectual contribution to the work, and approved it for publication.

## Conflict of Interest

The authors declare that the research was conducted in the absence of any commercial or financial relationships that could be construed as a potential conflict of interest.
